# Genotypic diversity alters invasive ability of *Hydrocotyle verticillata*

**DOI:** 10.3389/fpls.2025.1681443

**Published:** 2025-10-14

**Authors:** Yao-Le Ma, Wei-Long Li, Lin Huang, Wei Xue, Fei-Hai Yu

**Affiliations:** ^1^ Institute of Wetland Ecology & Clone Ecology, Taizhou University, Taizhou, Zhejiang, China; ^2^ School of Life and Environmental Sciences, Shaoxing University, Shaoxing, Zhejiang, China; ^3^ Zhejiang Key Laboratory for Restoration of Damaged Coastal Ecosystems, Taizhou University, Taizhou, Zhejiang, China; ^4^ Shaoxing University, Shaoxing, Zhejiang, China

**Keywords:** plant invasion, genetic diversity, plant diversity, complementarity effects, selection effects

## Abstract

Genetic diversity is a key component of biodiversity and plays an important role in shaping ecosystem structure and function. However, how genetic diversity of alien plants may influence their invasive ability requires further empirical investigation. In a greenhouse experiment, we planted a population of the alien plant *Hydrocotyle verticillata* consisting of 1, 2, 4 and 8 different genotypes in a native plant community to investigate the effects of genotypic diversity of alien plants on performance of both the alien plants and native plant communities. Shoot biomass of *H. verticillata* increased with increasing the number of genotypes, peaking at the 2-genotype treatment. By contrast, genotypic diversity did not influence the aboveground biomass or evenness of the native plant communities. Moreover, we found that the net genotypic diversity effects decreased with increasing the number of genotypes, and it was negatively correlated with the aboveground biomass of the native plant communities. These effects were attributed to the presence of particular genotypes in the mixed-genotype treatments. These results indicate that genotypic diversity of alien plants can alter their invasive ability through selection effects.

## 1 Introduction

With accelerating global changes and intensified human activities, alien plant invasions have become a major global ecological challenge (Díaz et al., 2019; Seebens et al., 2021; Chiu et al., 2023). Plant invasion can suppress native species and reduce biodiversity, alter community composition and weaken ecosystem functioning (Hejda et al., 2009; Liu et al., 2017; Vilà et al., 2006). Many hypotheses have been proposed to explain the success of alien plant invasions (Richardson and Pyšek, 2006), most of which emphasize the characteristics of invaders and the ecosystems they invade (Catford et al., 2009). Among these characteristics, genotypic diversity within the alien plant population has emerged as an important factor that may enhance their ability to establish and spread in novel environments (He et al., 2021; Hao et al., 2024). For instance, higher genotypic diversity can promote the invasive ability of *Spartina alterniflora* (Wang et al., 2012) and *Alternanthera philoxeroides* (Liu et al., 2022) due to enhanced resource utilization and stress tolerance. However, there is also evidence suggesting that high levels of genetic diversity are not a necessary condition for the successful introduction of plants (Rollins et al., 2013; Maebara et al., 2020). Their results suggest that the role of genetic diversity in invasion processes is not fully understood and may be context-dependent. This inconsistency highlights the need for further experimental studies that directly test how invader genotypic diversity influences its invasiveness in community settings.

Genetic diversity within an invasive plant population may influence its invasiveness through two primary mechanisms. First, higher genetic diversity may increase the likelihood of including a highly competitive genotype (i.e., selection effects; Hughes et al., 2008; Schoeb et al., 2015). If such a competitive genotype enhances population growth, genetic diversity may facilitate invasion success. Conversely, if this genotype suppresses overall population growth (e.g. through antagonistic interactions), increased genetic diversity could reduce plant invasions. Second, genetically diverse populations may contain genotypes with different ecological niches e.g., nutrient-acquisition strategies, which can lead to more complete resource utilization and greater potential for positive interactions (e.g., mutual facilitation) among genotypes. This complementarity effect can enhance population growth and competitive ability, thereby facilitating invasion (Fridley et al., 2007; Lankau and Strauss, 2007; Zerebecki and Hughes, 2023). While these mechanisms are theoretically well-supported, their relative contributions to the invasion success of genetically diverse populations remain poorly understood and require further empirical investigation.

*Hydrocotyle verticillata* Thunb. is a widespread invasive alien species in China (https://www.iplant.cn/ias). The successful invasion of this species can be attributed to its high phenotypic plasticity (Dong et al., 2015; Huang et al., 2022), positive plant-soil interactions (Begum et al., 2023; Xue et al., 2024), and considerable physiological adaptability (Kozeko et al., 2024). In the field, more than 20 genotypes of *H. verticillata* were identified and each population has 1 to 5 genotypes with often one genotype being exclusively dominant (Wang et al., 2020). Begum et al. (2022) found that increasing genotypic diversity of *H. verticillata* can promote population growth (Begum et al., 2022), but Huang et al. (2022) did not find evidence that genotypic diversity can alter productivity of *H. verticillata* population (Huang et al., 2022). Moreover, previous studies found that increasing the number of genotypes of *H. verticillata* population can promote the fluxes of CO_2_ (Zhu et al., 2023) and enhance NH_4_^+^-N uptake preference (Zhu et al., 2024); Liu et al. (2024) reported that increasing genetic diversity of *H. verticillata* population altered interspecific interactions between two subordinate species and facilitated their coexistence (Liu et al., 2024). Collectively, these results indicate that alterations in genotypic diversity within *H. verticillata* population may influence population structure and community−level dynamics. However, it remains unclear how genotypic diversity in *H. verticillata* influences its invasiveness within plant communities.

Here, we tested how genotypic diversity of the invasive plant *H. verticillata* may influence its invasive ability by planting populations of *H. verticillata* consisting of 1, 2, 4, and 8 genotypes with an experimental native plant community. We selected these four levels of genotypic diversity rather than the exact number of genotypes typically found in the wild (1–5 genotypes) because it is a widely used design in the diversity-ecosystem function experiment, and enables us to examine potential diversity effects beyond what is commonly observed, thereby providing insights into whether diversity effects plateau or reverse at higher levels. We tested the following hypotheses: (1) If genotypic diversity enhances the performance of *H. verticillata*, populations with greater genotypic diversity will produce higher biomass than those with lower genotypic diversity; (2) If genotypic diversity of *H. verticillata* increases its competitive impact, then native plant communities will grow worse when grown with *H. verticillata* populations of greater genotypic diversity compared to those with lower genotypic diversity.

## 2 Materials and methods

### 2.1 Experimental species and culture

*Hydrocotyle verticillata* Thunb. is a widely distributed species in temperate and tropical regions such as Europe, Southeast Asia, North America, and North Africa (http://www.iplant.cn/). It was introduced into China in the 1990s an ornamental garden plant but now is widely distributed in many habitats including wetlands, lawns, riverbanks, and ditches (Dong et al., 2015). This species exhibits high phenotypic plasticity and rapid reproductive growth, primarily through creeping stolons that produce roots and asexual offspring at each node (Xue et al., 2024). Its environmental adaptability and competitive ability are superior to many native species, potentially threatening native biodiversity and ecosystems (Qin et al., 2019; Begum et al., 2022).

In 2016, 128 ramets of *H. verticillata* were collected from 10 different sites in southern China (Appendix: **Supplementary Figure S1**). A total of 20 genotypes were identified using AFLP based on genomic DNA (Wang et al., 2020), and ramets of different genotypes were cultivated in separate containers in a common garden. On October 10, 2021, we collected ramets from 10 genotypes for plant cultivation. Each ramet had a node with some adventitious roots, a petiole of 2 cm long, a proximal, and a distal internode of 1 cm long. We planted each ramet in a small pot filled with potting soil (nitrogen: 0.14 g L^−1;^ phosphorus: 0.10 g L^−1^; potassium: 0.18 g L^−1^). After three weeks, we selected ramets of similar size for the experiment below. The height and biomass of the initial ramets were 6.70 ± 2.88 cm (mean ± SE, n = 10) and 0.018 ± 0.007 g (mean ± SE, n = 10), respectively.

We used 10 native plants in the experiment (**Appendix: Supplementary Table 1**). These species were chosen because they represent common taxa in local plant communities, particularly in transitional habitats where terrestrial and aquatic species may coexist (http://www.iplant.cn/). The native status of *Nepeta cataria* remains to be debated (https://powo.science.kew.org/taxon/452333-1), but we treated it as a native species in this study (Ibrahim et al., 2022). On October 10, 2021, we evenly sowed seeds of each plant in a germination tray filled with potting soil. The seeds were purchased from a seed company (Hengkaimaoyi, Hangzhou, Zhejiang Province, China), which provides seeds collected from wild populations (in Hangzhou, Zhejiang Province) rather than inbred or artificially selected lines. Daily water was added to each tray. On November 1, 2021, three-week-old seedlings with similar size were selected and used in the experiment described below.

### 2.2 The experiment

**Figure 1:**
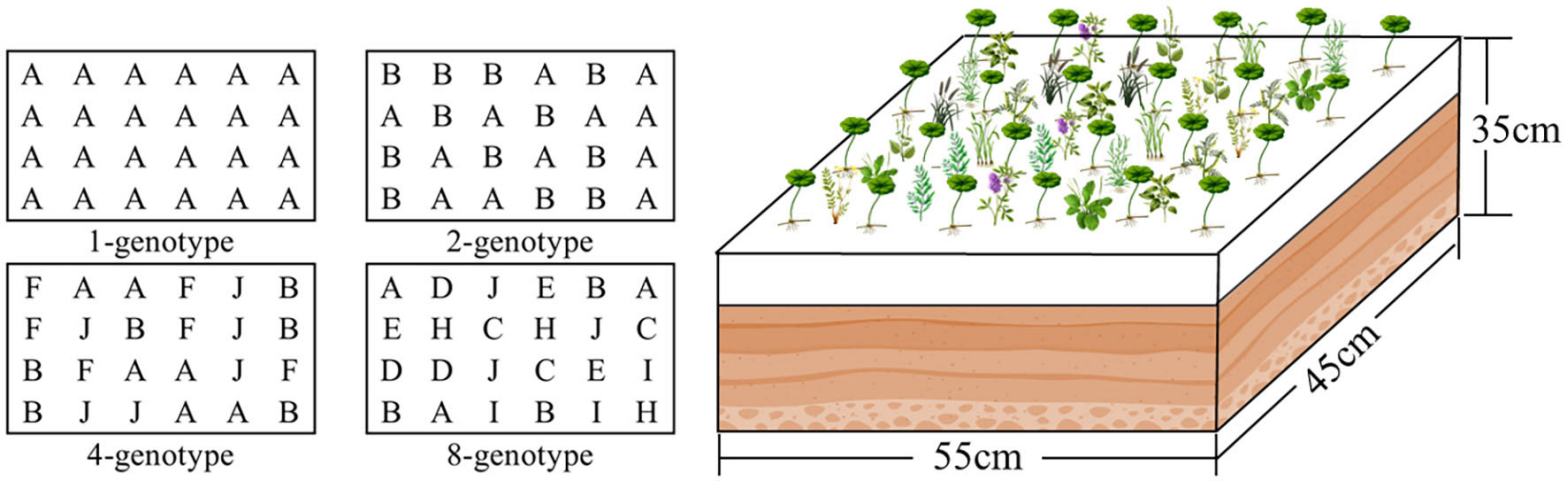
Schematic diagram of the experimental design. Populations of the alien plant *Hydrocotyle verticillata* with different levels of genotypic diversity (1, 2, 4, or 8 genotypes) were grown together with a native plant community of 10 species. Each container had 24 planting positions allocated for *H. verticillata*, with letters (A-J) representing the 10 different genotypes and their corresponding planting positions. The number of ramets per genotype varied with diversity level: 24 (1-genotype), 12 (2-genotype), 6 (4-genotype), or 3 (8-genotype). Round-leaf symbols represent *H. verticillate*, while other shapes represent native species. Thirty native seedlings were randomly planted at 30 evenly spaced positions in each container.

We established 60 experimental containers (55 cm length × 45 cm width × 35 cm height), each filled with a 2:1:1 (v: v: v) mixture of peat, soil and river sand to a depth of 15 cm. These containers were assigned to four levels of *H. verticillata* genotypic diversity, i.e., 1, 2, 4, and 8 genotypes. In the 1-genotype treatment, each of the 10genotypes was grown in monoculture (24 ramets; **Figure 1**), with three replicate containers per genotype (30 containers in total). In the 2-genotype treatment, we created 10 unique two-genotype combinations, and each combination was planted in one container with 12 ramets per genotype (24 ramets in total; **Figure 1**); These combinations were selected from the pool of 10 genotypes such that each genotype appeared exactly twice across the 10 containers. Similarly, in the 4- and 8-genotype treatments, we created 10 unique four-genotype and eight-genotype combinations, and each combination was planted in one container with 6 and 3 ramets per genotype, respectively (24 ramets in total; **Figure 1**); These combinations were also selected from the pool of 10 genotypes such that each genotype appeared exactly four and eight times across the 10 containers, respectively. This design resulted in 60 containers in total: 30 for the 1-genotype treatment (10 genotypes × 3 replicates) and 10 each for the 2-, 4-, and 8-genotype treatments. These unique genotype combinations used are listed in **Appendix: Supplementary Table 2**.

As our study was designed specifically to examine how genotypic diversity of *Hydrocotyle verticillata* influences its invasive ability within the context of native communities, we introduced a plant community consisting of the 10 native species into all *H. verticillata* populations. On November 15, 2021, when the *H. verticillata* population was established, we transplanted three seedlings of each native plant into each container. The 30 seedlings were randomly planted at 30 evenly distributed points in the experimental container (**Figure 1**). Dead seedlings were replaced in the first week after transplanting.

The experiment lasted for nine months (from November 1, 2021, to July 24, 2022). During this experiment, all pots were watered regularly and fertilized twice (N: P: K = 14:14:14). The daily average air temperature is 19.0 °C and the air humidity is 76.8%.

At the end of this experiment, we harvested the shoots of *H. verticillata* and each of the native plants separately. When harvested, *Setaria viridis*, *Potentilla chinensis* and *Astragalus membranaceus* were completely competitive excluded across all treatment containers. All plant materials were oven-dried at 70°C for at least 48 hours.

### 2.3 Data analysis

To evaluate the invasive ability of *H. verticillata*, we first used a linear mixed-effects model to test the effect of genotypic diversity (1-, 2-, 4- vs. 8-genotype) on shoot biomass of *H. verticillata*. In this model, genotypic diversity of *H. verticillata* was included as a fixed effect, and identity of combinations was used as a random effect. A Tukey test was used for *post-hoc* comparisons when a significant effect was detected.

To examine the effect of *H. verticillata* on the native plant community, we first calculated total aboveground biomass of the community by summing the shoot biomass of each component species. We also calculated the evenness index as: $H=H^{\prime }/\ln S$ , where *H’* is the Shannon-Wiener diversity index, and *S* is the number of plant community species. This index was used to quantify the relative distribution of abundances among species in the community, reflecting community structure and should not be interpreted as a direct measure of seedling survival or mortality. Then, we used a linear mixed-effects model to test the effect of genotypic diversity of *H. verticillata* on the aboveground biomass and evenness of the native plant community. In this model, genotypic diversity of *H. verticillata* was included as a fixed effect, and identity of combinations was used as a random effect. A Tukey test was used for *post-hoc* comparisons when a significant effect was detected.

To explore the underlying mechanisms for these effects, we conducted a two-step analysis. First, we calculated the net genotypic diversity effect (NE) of *H. verticillata* as: $NE=Y_{obs}-\sum_{i=1}^{S}p_{i}M_{i}$ , where *Y_obs_* is the observed shoot biomass of a multi-genotype population (2, 4, or 8 genotypes), *S* is the number of genotypes in the mixture, *p_i_* is the proportion of genotype *i* planted in the mixture, and *M_i_* is the mean shoot biomass of genotype *i* grown in monoculture. We used *t*-tests to test whether the net genotypic diversity effect at each genotypic diversity level differed significantly from zero, and linear mixed-effects model followed by Tukey tests to test the difference in the net effect of genotypic diversity among different genotypic diversity treatments (2- vs. 4- vs. 8-genotype treatments). We also used linear regression to test whether the net genotypic diversity effect of *H. verticillata* influenced the aboveground biomass of the native plant community. Then, we conducted a one-way ANOVA followed by Tukey tests to assess the difference in shoot biomass of *H. verticillata* and aboveground biomass of the native plant community among mono-genotype treatments. We also used two-sample *t*-tests to examine whether the presence of a particular genotype in the 2-, 4- and 8-genotype treatments can influence shoot biomass of *H. verticillata* and aboveground biomass of the native plant community.

All analyses were performed using R (4.4.0). The linear mixed-effects models were fitted using package *nlme* (3.1-166). Tukey tests were conducted using the *glht* function in the *multcomp* package (1.4-26). All data were checked graphically for normality and homogeneity of variance.

## 3 Results

**Figure 2:**
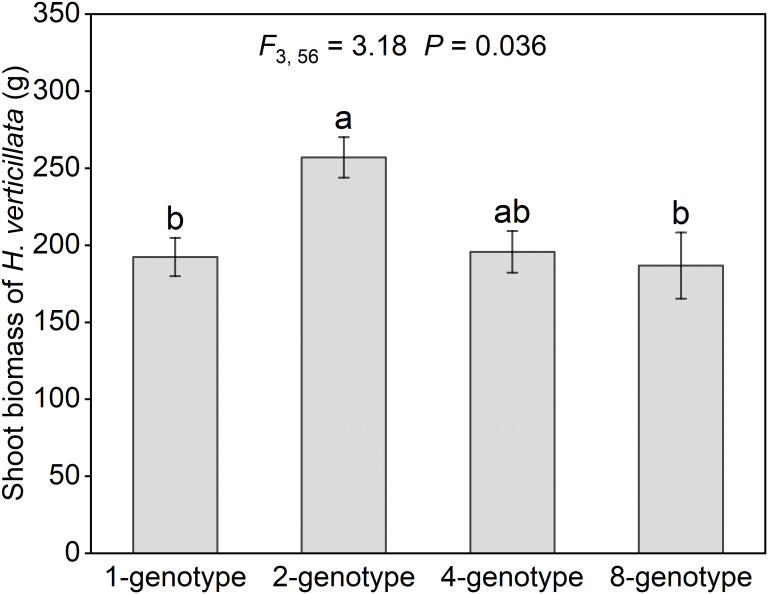
Shoot biomass of the alien plant *Hydrocotyle verticillata* with different levels of genotypic diversity in the community. Mean values and standard errors are presented; different lowercase letters (a-b) indicate significant differences (*P*<0.05). *F*- and *P*-values based on linear-mixed effects models are presented.

The invasive plant *H. verticillata* showed a hump-shaped response to genotypic diversity (**Figure 2**). Shoot biomass in 2-genotype treatment was significantly higher than in 1- and 8-genotype treatments, but did not differ from that of the 4-genotype treatment (**Figure 2**). No significant differences were detected among the 1-, 4- and 8-genotype treatments (**Figure 2**). These results suggest that intermediate genotypic diversity can enhance invasive performance. For the native plant communities, neither aboveground biomass nor evenness differed significantly among the four genotypic diversity treatments (**Appendix: Supplementary Figure S2**). The species-specific analysis showed that the majority of native species did not show significant differences among the four genotypic diversity treatments, with the exception of *Medicago sativa*, which exhibited increased biomass with increasing genotypic diversity (**Appendix: Supplementary Figure S3**).

**Figure 3:**
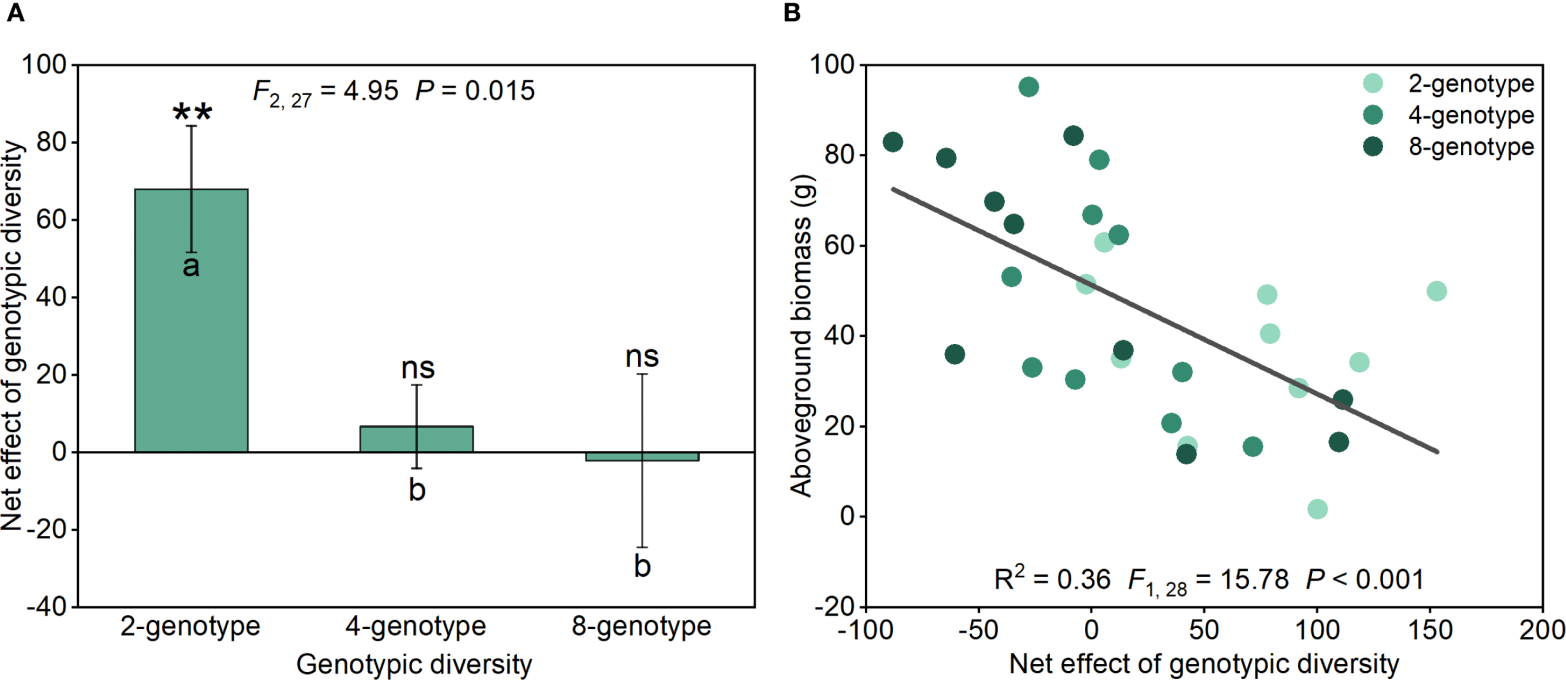
Net genotypic diversity effect of the alien plant *Hydrocotyle verticillata* under different treatments **(A)** and regression analysis of the net genotypic diversity effect of *Hydrocotyle verticillata* with the aboveground biomass of the native plant community **(B)**. Mean values and standard errors are presented; different lowercase letters **(A, B)** indicate significant differences (*P*<0.05). Symbols: ^ns^ *P* > 0.05, and ^**^ *P*<0.01 (one-sample t-tests). *F*-, *P*- and *R* ^2^-values based on linear regressions are presented.

Net genotypic diversity effect of *H. verticillata* in the 2-genotype treatment was significantly greater than in the 4- and 8-genotype treatments (**Figure 3A**). The net genotypic diversity effect was positive in the 2-genotype treatment, but there was no significant net genotypic diversity effect in the 4- and 8-genotype treatments (**Figure 3A**). Importantly, aboveground biomass of the native plant communities declined significantly with increasing net genotypic diversity effect of *H. verticillata* (**Figure 3B**), indicating that stronger positive net genotypic diversity effects in the invader were associated with reduced productivity in the native community.

**Figure 4:**
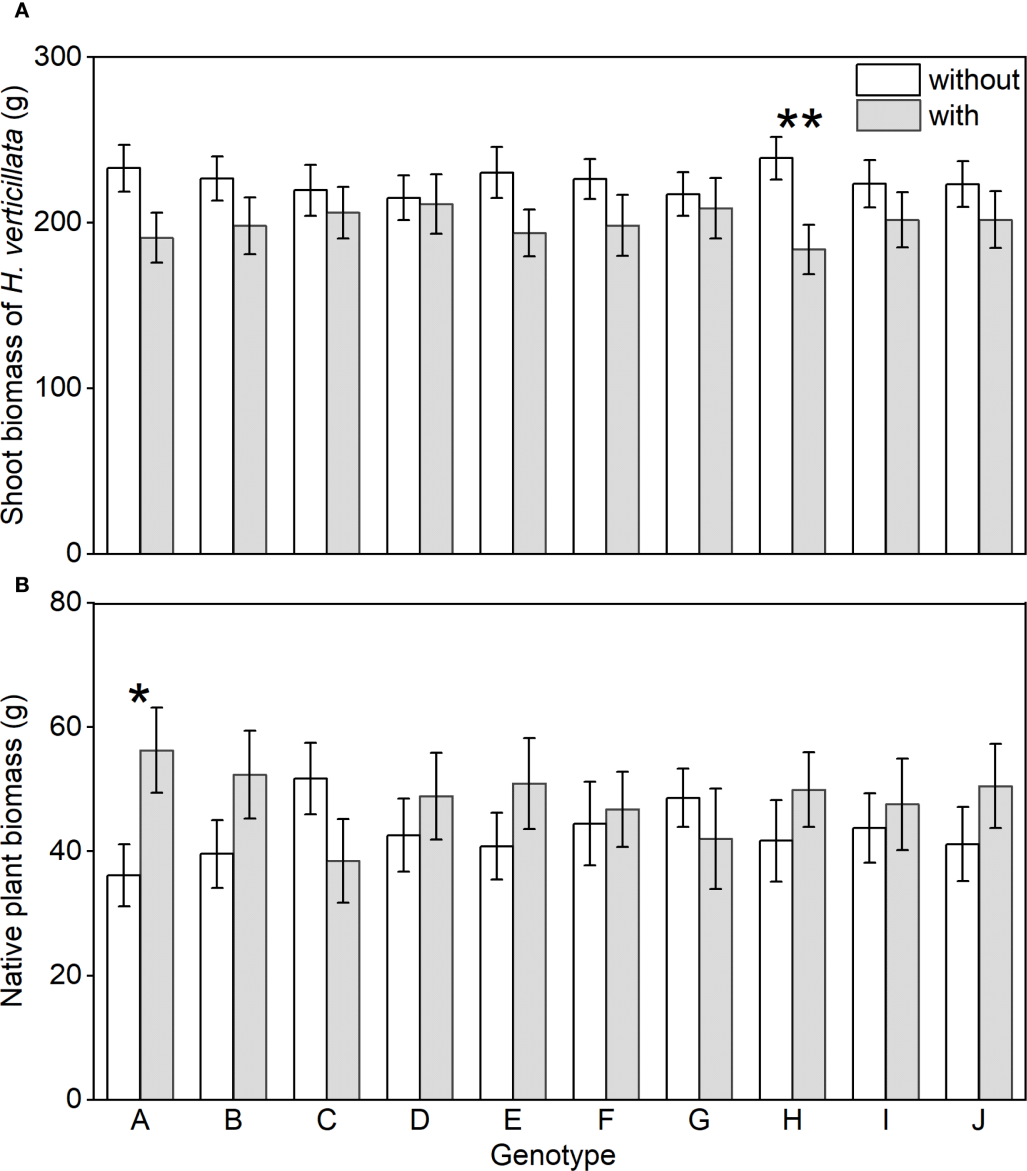
Shoot biomass of the alien plant *Hydrocotyle verticillata* **(A)** and aboveground biomass of the native community **(B)** in the treatment with and without the presence of the target genotypes. Mean values and standard errors are presented. Symbols: ^*^ *P<* 0.05, and ^**^ *P*<0.01 (two-sample t-tests).

We found no significant differences in shoot biomass of *H. verticillata* or native community biomass among the mono-genotype treatments (**Appendix: Supplementary Figure S4**). However, particular genotypes influenced outcomes in mixed-genotype treatments. The presence of genotype A in mixed-genotype treatments significantly increased native community biomass (**Figure 4B**); the presence of genotype H in mixed-genotype treatments significantly suppressed invasive biomass (**Figure 4A**). Other genotypes showed no significant effects on either invasive or native biomass (**Figure 4**). Together, these findings indicate that specific genotypes can disproportionately shape invasion outcomes within mixed-genotype populations.

## 4 Discussion

Our results show that growth of the alien plant *H. verticillata* was highest in the 2-genotype treatment. Although we did not observe evidence that genotypic diversity of *H. verticillata* can influence growth of the native plant communities, growth of the native plant communities was negatively correlated to net genotypic diversity effects of *H. verticillata*. These effects are likely driven by the presence of particular genotypes, as reflected by the significant influence of specific genotypes within multi-genotype treatments on the shoot biomass of both the alien plant and the native communities. These results indicate that genotypic diversity of the alien plant *H. verticillata* can influence its invasiveness due to the presence of specific genotypes. These findings have important contributions for the understanding of the ecological mechanisms of alien plant invasions, particularly the role of genotypic diversity. Therefore, limiting the introduction and mixing of multiple genotypes, or targeting low-diversity populations in restoration efforts could potentially offer novel strategies for the management and control of invasive plants.

### 4.1 Effects of genotypic diversity on growth of the alien plant *H. verticillata*

Many studies have revealed that genetic diversity can improve population productivity (Hughes et al., 2008; Crutsinger et al., 2013; Begum et al., 2023). In consistence with these studies, we also found that 2-genotype mixtures of *H. verticillata* had greater population growth than 1-genotype populations. Similar to plant species diversity effects, the positive genotypic diversity effects can be attributed to complementarity effects and selection effects (Loreau and Hector, 2001; Brown and Rice, 2010; Hector et al., 2010; Larkin et al., 2023). Genetic diversity affects the viability and adaptive potential of alien species, as different genotypes may differ in their abilities and preferences to utilize resources (e.g. light, water, nutrients; Leishman and Thomson, 2005; Lankau and Strauss, 2007; Fridley and Grime, 2010), increasing genotypic diversity may result in a more comprehensive utilization of resources, contributing to increased productivity of the population (Zeleny, 2007; Agashe, 2009; Wang et al., 2012; Tang et al., 2022). Moreover, it is also possible that in the population with higher genotypic diversity, there is a higher likelihood to include productive genotypes, contributing to the higher population growth. Unfortunately, we were unable to distinguish ramets of different genotypes at harvest, so we failed to directly measure the complementarity effects and selection effects.

However, the positive genotypic diversity effects were not maintained at higher diversity levels (4 and 8 genotypes). At higher diversity levels, the probability of including genotypes with negative effects may increase. Our genotype-level analysis did show that the presence of genotype H in the multi -genotype treatments decreased the growth of the invasive populations, providing indirect evidence that the selection effects may have played a key role in regulating the population performance of *H. verticillata.*

### 4.2 Effects of genotypic diversity on growth of the native plant communities

Most studies have shown that plant invasions will lead to significant reduction in native plant community diversity and productivity (Winter et al., 2009; Castro-Diez et al., 2016; Livingstone et al., 2020). However, in the current study, we did not find evidence that genotypic diversity of the alien plant *H. verticillata* can alter productivity and evenness of the native plant communities. One possible explanation is that species composition and niche of the native plant community were relatively stable, and species composition may regulate the resource competition through the interactions between species and niche differentiation, thus weakening the influences of genotypic diversity (Vellend, 2006; Hart et al., 2019; Brandt et al., 2023).

However, productivity of the native plant communities was negatively correlated with the net genotypic diversity effect of *H. verticillata*, which was positive only in the 2-genotype treatments and disappeared at higher diversity levels (**Figure 3**). Our genotype-level analysis further suggested that the presence of genotype H in the mixed-genotype treatments may reduce the growth of the alien plant population and thus promote the growth of native plant communities. Therefore, genotypic diversity of the alien plants may inhibit its invasion success if particular genotypes present in the communities, which has important implications for the control of plant invasions.

## 5 Conclusions

We conclude that genotypic diversity of the alien plant *H. verticillata* can alter its invasive ability by changing growth of both the invaded native plant communities and itself due to the presence of particular genotypes. These results indicate that selection effects may play a key role in regulating invasion processes. However, these effects were found within the context of native communities varying in genetic diversity, we cannot exclude the potential confounding effects of native communities and their genetic diversity on the invasive ability of *H. verticillata*. Future studies testing genotypic diversity-invasive ability relationships should consider a gradient of species diversity and genetic diversity of native communities. Despite that, our results highlight that it could potentially offer new strategies and methods for the management and control of invasive plants through manipulating genotypic diversity.

## Data Availability

The raw data supporting the conclusions of this article will be made available by the authors, without undue reservation.

## Supplementary material

Supplementary Figure 1 Locations of the 10 sites where the initial ramets of *Hydrocotyle verticillata* were collected.

Supplementary Figure 2 Aboveground biomass **(A)** and evenness **(B)** of the native plant community under the treatments with different genotypic diversity of the alien plant *Hydrocotyle verticillata*. Mean values and standard errors are presented. *F*- and *P*-values based on linear-mixed effects models are presented.

Supplementary Figure 3 Aboveground biomass of Cynodon dactylon **(A)**, Achyranthes bidentata **(B)**, Nepeta cataria **(C)**, Plantago asiatica **(D)**, Leonurus japonicus **(E)**, Pennisetum alopecuroides **(F)**, and Medicago sativa **(G)** within the community under the treatments with different genotypic diversity of the alien plant Hydrocotyle verticillata. F- and P-values based on linear-mixed effects models are presented. Different lowercase letters **(a-b)** indicate significant differences (P<0.05).

Supplementary Figure 4 Shoot biomass of the alien plant *Hydrocotyle verticillata* **(A)** and aboveground biomass of the native community **(B)** among mono-genotype treatments. Mean values and standard errors are presented. *F*- and *P*-values based on one-way ANOVAs are presented.

Supplementary Table 1 Species used in the plant community.

Supplementary Table 2 Genotype combination used for different genotypic diversity levels.
