# Supplementary material for: Genotypic diversity alters invasive ability of Hydrocotyle verticillata
Source: Front Plant Sci. 2025 Oct 14;16:1681443. doi: 10.3389/fpls.2025.1681443 (PMC12558809; doi:10.3389/fpls.2025.1681443)

Appendix

Table 1 Species used in the plant community

Latin name	Family name	Life form	Functional group	Status	Typical habitats
Cynodon dactylon	Poaceae	perennial	grass	noninvasive	roadsides, field margins, riverbanks
Setaria viridis	Poaceae	annual	grass	noninvasive	hill slopes, roadsides, grasslands
Pennisetum alopecuroides	Poaceae	perennial	grass	noninvasive	grassy hillsides, roadsides, field margins
Leonurus japonicus	Lamiaceae	annual / biennial	forb	noninvasive	sunny areas
Plantago asiatica	Plantaginaceae	biennial / perennial	forb	noninvasive	mountain slopes, ravines, riverbanks, fields, roadsides
Nepeta cataria	Lamiaceae	perennial	forb	noninvasive	thickets, around houses
Achyranthes bidentata	Amaranthaceae	perennial	forb	noninvasive	hillsides
Potentilla chinensis	Rosaceae	perennial	forb	noninvasive	forest margins, thickets, meadows, grasslands, ravines
Astragalus membranaceus	Fabaceae	perennial	legume	noninvasive	forest margins, thickets, meadows, mountain slopes
Medicago sativa	Fabaceae	perennial	legume	noninvasive	roadsides, fields, grasslands, stream banks

Information is based on the Flora of China (http://www.iplant.cn/).

Table 2 Genotype combination used for different genotypic diversity levels

	Genotypic diversity																																							
Genotype ID	1										2										4										8									
A(JX-22)	■													■			■					■	■	■			■				■	■	■	■	■	■		■	■	
B(WH-9)		■											■				■							■		■		■	■			■		■	■	■	■	■	■	■
C(WZ-7)			■								■				■						■					■	■	■			■	■	■		■		■	■	■	■
D(WH-1)				■								■		■							■	■			■				■		■	■	■	■		■	■		■	■
E(HZ-13)					■										■					■	■		■		■				■		■		■	■	■		■	■	■	■
F(TZ-1)						■						■							■					■			■	■		■		■	■	■	■	■	■	■		■
G(HZ-5)							■				■							■				■	■		■	■					■	■		■	■	■	■	■		■
H(CQ-2)								■								■			■		■						■	■		■	■		■	■	■	■	■		■	■
I(TZ-8)									■				■					■				■				■			■	■	■	■	■	■		■	■	■	■	
J(NB-3)										■						■				■			■	■	■					■	■	■	■		■	■		■	■	■
Replicate[1]	1	1	1	1	1	1	1	1	1	1	1	1	1	1	1	1	1	1	1	1	1	1	1	1	1	1	1	1	1	1	1	1	1	1	1	1	1	1	1	1

[1] Each population had one replicate for each genotype combination. Therefore, there were 10 (1 × 10 genotypes) replicates for monocultures, 10 (1 × 10 combinations) replicates for the 2-genotype, 4-genotype and 8-genotype mixtures.

Figure S1 Locations of the 10 sites where the initial ramets of *Hydrocotyle verticillata* were collected.

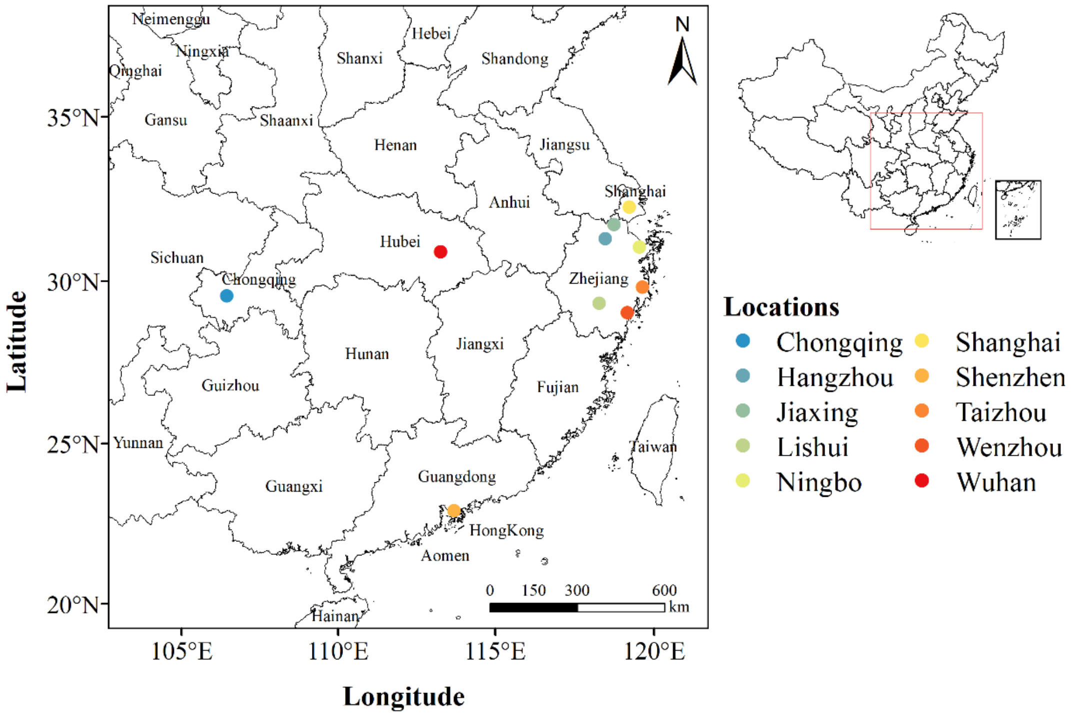

Figure S2 Aboveground biomass (A) and evenness (B) of the native plant community under the treatments with different genotypic diversity of the alien plant *Hydrocotyle verticillata*. Mean values and standard errors are presented. *F*- and *P*-values based on linear-mixed effects models are presented.

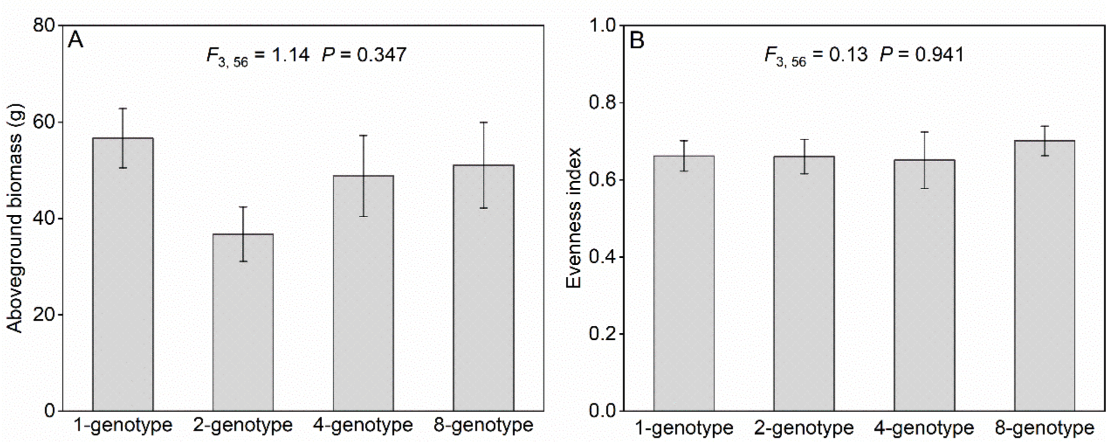

Figure S3 Aboveground biomass of *Cynodon dactylon* (A), *Achyranthes bidentata* (B), *Nepeta cataria* (C), *Plantago asiatica* (D), *Leonurus japonicus* (E), *Pennisetum alopecuroides* (F), and *Medicago sativa* (G) within the community under the treatments with different genotypic diversity of the alien plant *Hydrocotyle verticillata*. *F*- and *P*-values based on linear-mixed effects models are presented. Different lowercase letters (a-b) indicate significant differences ($P < 0.05$).

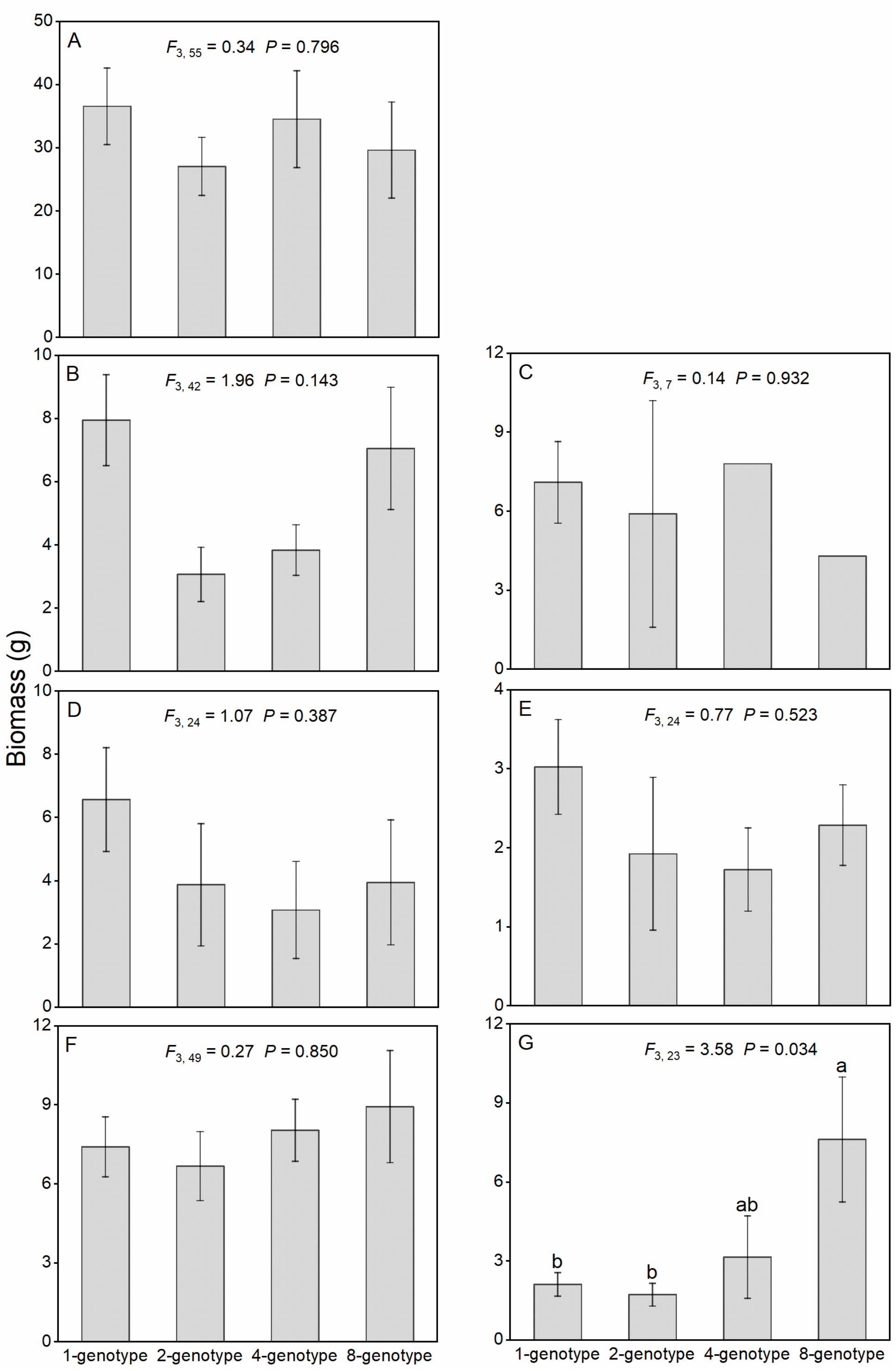

Figure S4 Shoot biomass of the alien plant *Hydrocotyle verticillata* (A) and aboveground biomass of the native community (B) among mono-genotype treatments. Mean values and standard errors are presented. *F*- and *P*-values based on one-way ANOVAs are presented.

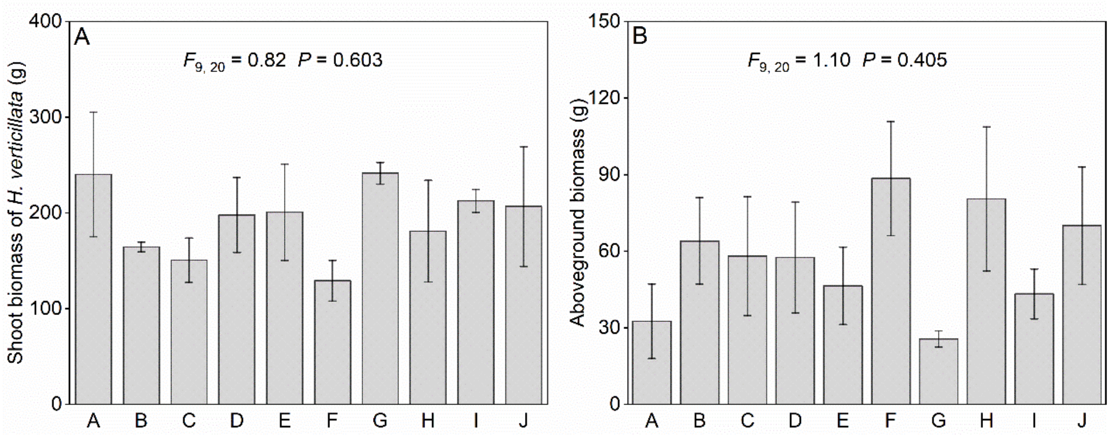

Supplement: Supplementary Figure 1 — Locations of the 10 sites where the initial ramets of Hydrocotyle verticillata were collected. [file DataSheet1.pdf]
